# The King's Fund and the Bill

**Published:** 1911-07-22

**Authors:** 


### HOSPITALS AND STATE INSURANCE.

### THE KING'S FUND AND THE BILL.

The Executive Committee of King Edward's Hospital Fund for London recently appointed a sub-committee to inquire into the probable effect of the National Insurance Bill upon the hospitals receiving grants from the fund. The sub-committee ascertained the views of thirty-two of the principal London hospitals and drew up a report thereon. Upon receiving this report the Executive Committee agreed upon the following statement, which represents an abstract of the views of these hospitals or their secretaries. The divergent nature of the replies makes it evident that any statement as to the probable effect of the Bill must be largely speculative.

### How the Bill will affect Hospitals.

As the Bill now stands the insurance scheme does not apply to certain classes, nor, in the earlier years of its operation, to the families and dependents of the insured. One of the largest of these hospitals estimates that between 60 per cent. and 70 per cent. of the persons now treated in that hospital will be unaffected by the Bill.

It further appears from the replies that the balance of opinion amongst these hospitals inclines in the following directions:—

1. That as regards the number of in-patients it is anticipated that the effect, if any, will be rather to increase the total numbers, even though the proportion of the tuberculous cases may be eventually reduced.

2. That as regards out-patients it is anticipated that a different class of out-patient may have to be dealt with, and that the effect on numbers, if any, will be towards reduction.

3. That there is widespread apprehension that the contributions from employers and employed will be reduced.

4. That Clause 15—" Administration of sanatorium benefit "—will not, it is thought, have any considerable effect in the relief of general hospitals.

5. That Clause 17, " Power to approved societies to subscribe to hospitals, etc.," is not thought likely to operate beneficially if such contribution is optional, and, if compulsory, there is some apprehension lest this might lead to intervention in management.

6. That while complete statistics are not available as to the numbers of members of Friendly Societies who at present enjoy the benefits of hospital treatment, it appears that a considerable proportion of adult male patients at these hospitals are members of Friendly Societies or clubs; but that little contribution to these hospitals is at present received from such societies.

### Conclusions.

The Executive Committee points out that while under Clause 8, as amended, the Bill purports to confer on insured persons full medical treatment and attendance, including medicines and surgical appliances, the only means for supplying such benefits in non-tuberculous cases—apart from optional contributions of approved societies to hospitals and charitable institutions (Clause 17)—appears to be by arrangements made with individual medical practitioners (Clause 14, 1).

The Executive Committee having given due regard to the replies received from the hospitals, shares the opinion generally held that under such arrangements the utilisation of hospitals for more serious cases is not likely to diminish and may, not improbably, increase, with corresponding effect on expenditure; and that the Bill in its present form will tend rather to the reduction than to the augmentation of the hospital's revenue.

